# Leukocyte Telomere Length Predicts Severe Disability in Relapsing-Remitting Multiple Sclerosis and Correlates with Mitochondrial DNA Copy Number

**DOI:** 10.3390/ijms24020916

**Published:** 2023-01-04

**Authors:** Gabriela del Carmen López-Armas, Martha Eloisa Ramos-Márquez, Mónica Navarro-Meza, Miguel Ángel Macías-Islas, Ana Miriam Saldaña-Cruz, Abraham Zepeda-Moreno, Fernando Siller-López, José Alfonso Cruz-Ramos

**Affiliations:** 1 Laboratorio de Biomédica-Mecatrónica, Subdirección de Investigación y Extensión, Centro de Enseñanza Técnica Industrial Plantel Colomos, Guadalajara 44638, Mexico; 2 Departamento de Biología Molecular y Genómica, Centro Universitario de Ciencias de la Salud, Universidad de Guadalajara, Guadalajara 44340, Mexico; 3 Laboratorio C. de Neuronutrición y Memoria, Departamento de Promoción, Preservación y Desarrollo de la Salud, Centro Universitario del Sur, Universidad de Guadalajara, Ciudad Guzmán 49000, Mexico; 4 Departamento de Neurociencias, Centro Universitario de Ciencias de la Salud, Universidad de Guadalajara, Guadalajara 44340, Mexico; 5 Departamento de Fisiología, Centro Universitario de Ciencias de la Salud, Universidad de Guadalajara, Guadalajara 44340, Mexico; 6 Departamento de Clínicas de la Reproducción Humana, Centro Universitario de Ciencias de la Salud, Universidad de Guadalajara, Guadalajara 44340, Mexico; 7 Programa de Bacteriología, Facultad de Ciencias de la Salud, Universidad Católica de Manizales, Manizales 170002, Colombia; 8 Departamento de Clínicas Médicas, Centro Universitario de Ciencias de la Salud, Universidad de Guadalajara, Guadalajara 44340, Mexico; 9 Coordinación de Investigación, Instituto Jalisciense de Cancerología, Guadalajara 44280, Mexico

**Keywords:** leukocyte telomere length, mitochondrial DNA, relapsing-remittent multiple sclerosis, disability, aging

## Abstract

Multiple sclerosis (MS) is a chronic autoimmune inflammatory disease that affects the nervous system. Peripheral blood leukocyte telomere length (LTL) and mitochondrial DNA copy number (mtDNA-CN) are potential biomarkers of neurological disability and neural damage. Our objective was to assess the LTL and mtDNA-CN in relapsing-remitting MS (RRMS). We included 10 healthy controls, 75 patients with RRMS, 50 of whom had an Expanded Disability Status Scale (EDSS) from 0 to 3 (mild to moderate disability), and 25 had an EDSS of 3.5 to 7 (severe disability). We use the Real-Time Polymerase Chain Reaction (qPCR) technique to quantify absolute LTL and absolute mtDNA-CN. ANOVA test show differences between healthy control vs. severe disability RRMS and mild-moderate RRMS vs. severe disability RRMS (*p* = 0.0130). LTL and mtDNA-CN showed a linear correlation in mild-moderate disability RRMS (r = 0.378, *p* = 0.007). Furthermore, we analyzed LTL between RRMS groups with a ROC curve, and LTL can predict severe disability (AUC = 0.702, *p* = 0.0018, cut-off < 3.0875 Kb, sensitivity = 75%, specificity = 62%), whereas the prediction is improved with a logistic regression model including LTL plus age (AUC = 0.762, *p* = 0.0001, sensitivity = 79.17%, specificity = 80%). These results show that LTL is a biomarker of disability in RRMS and is correlated with mtDNA-CN in mild-moderate RRMS patients.

## 1. Introduction

Multiple sclerosis (MS) is a chronic autoimmune disease of multifactorial origin (genetic susceptibility and environmental factors) characterized by heterogeneous neurological dysfunction and clinical features secondary to damage to the central and peripheral nervous systems [1,2,3]. Globally, MS is the leading non-traumatic cause of neural disability in young adults, affecting approximately 2.8 million people [4]. Demyelination and axonal loss in the central nervous system (CNS), mediated by an inflammatory and a subsequent neurodegenerative phase, are crucial for the episodic and irreversible progression of MS [5]. Initially, MS patients may have mild psychological and cognitive impairments which progress over time to severe neurological and motor limitations [6,7]. These MS neurodegenerative changes are assessed using the Expanded Disability Status Scale (EDSS) based on well-defined clinical and imaging parameters. According to this scale, patients are graded from 0 (no signs, no symptoms) to 10 (death) [8,9,10,11]. MS patients can have different phenotypes: (1) primary progressive multiple sclerosis (PPMS), which manifests episodes of neurological dysfunction, is progressive, and without recovery; (2) relapsing-remitting multiple sclerosis (RRMS), characterized by episodes of neurological dysfunction with full or partial recovery, and (3) secondary progressive multiple sclerosis (SPMS), which results from RRMS and acquires a similar pattern of progressive dysfunction to PPMS without recovery [12]. The mechanisms of neuroinflammation in MS resemble those of aging, but in patients with MS, they appear to be activated earlier in life, exhibit greater intensity, and ultimately affect the course of the disease [13]. It has been described that the central molecular mechanisms that regulate aging are shared with the pathogenesis of most chronic-degenerative diseases, such as cellular inflammation, mitochondrial damage, and telomere size shortening, mediated mainly by oxidative stress generated by reactive oxygen species (ROS). Therefore, the study of these common pathways is of current interest in MS research [14].

The telomere implication with aging relies on the fact that telomeres are nucleoprotein structures that protect the ends of chromosomes, prevent detrimental structural changes such as intrachromosomal fusion, provide genomic stability, and regulate cellular senescence [15,16]. Telomere shortening has been associated with MS as independent of age and correlated with more significant disability, lower brain volume, higher recurrence rate, and shorter conversion time from relapsing to progressive MS [17,18,19]. There is strong evidence that telomere measurement in leukocytes is a useful tool to be considered as a biomarker in the future since leukocytes are a niche of hematopoietic cells that circulate throughout the body and are sensitive to pathophysiological changes that occur over time [20].

Another key factor associated with cellular aging and neurodegeneration in MS is mitochondrial deficiency [21,22]. Analyses of mitochondrial DNA (mtDNA) in MS showed a significant decrease in mitochondrial DNA copy number (mtDNA-CN) in cerebrospinal fluid and lymphocytes of MS patients compared with control subjects [22,23]. Also, the study by Al-Kafaji et al. (2020) showed a significant decrease in mtDNA-CN in RRMS patients with more than ten years of MS diagnosis compared with those patients with less than ten years of diagnosis [22].

In the present study, we aim to evaluate mtDNA-CN and leukocyte telomere length (LTL) as prognostic biomarkers of severe disability in RRMS patients. Research on the link between aging and neurodegeneration may help classify MS patients based on their current disease status or prognosis and may lead to better therapeutic strategies to prevent accelerated aging and deterioration of MS.

## 2. Results

### 2.1. Patients

All participants are Mexican mestizos with Mexican parents [24]. Patients were randomly selected from the local RRMS cohort. The sex distribution, mean age, and clinical phenotypes of patients are similar to that observed in western Mexico for RRMS [25]. Healthy controls were obtained from the general population. The age was 40.6 ± 8.73, and there was no statistical difference vs. RRMS patients. Demographic and treatment data of the 75 patients are presented in Table 1. In the RRMS patients, the age was 39.4 ± 11.5, the years since diagnosis was 8.60 ± 5.88, the progression rate was 0.67 ± 0.83, and the EDSS 2.9 ± 1.71. There was no significant difference in sex, age, or time since diagnosis between groups. There were significant differences regarding progression rate (progression rate = EDSS/year with the disease) * *p* = 0.00145 and EDSS * *p* = 0.00001. Most RRMS patients (50%) were treated with Glatiramer Acetate; the second drug was Interferon β with 25%.

### 2.2. LTL and mtDNA-CN in Healthy Controls, Mild-Moderate Disability, and Severe Disability

**Figure 1:**
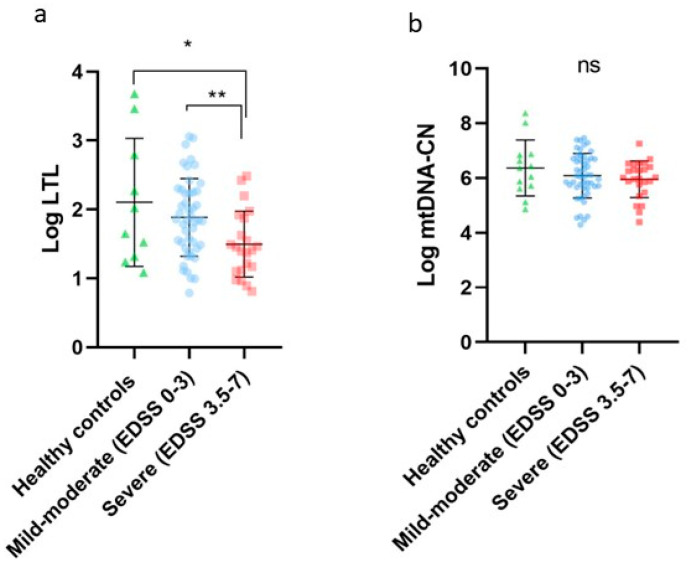
(**a**) Log LTL in RRMS patients; healthy controls (green), mild-moderate disability EDSS 0–3 (blue), and severe disability EDSS 3.5–7 (red). * *p* = 0.0094; ** *p* = 0.0140, and one-way ANOVA *p* = 0.0130. (**b**) Log mtDNA-CN in RRMS patients: healthy controls (green), mild-moderate disability (blue), and severe disability (red) *p* = 0.337. (ns) not significant.

LTL in the healthy controls group was 5.26 Kb, the mild-moderate disability group (EDSS 0–3) was 3.98 Kb, and the severe disability group (EDSS 3.5–7) was 2.98 Kb. For comparisons of Log LTL (2.10 ± 0.92 vs. 1.88 ± 0.56 vs. 1.49 ± 0.47) and Log mtDNA-CN (6.36 ± 1.02 vs. 6.08 ± 0.81 vs. 5.95 ± 0.66) between healthy controls, mild-moderate disability (EDSS 0–3) and severe disability (EDSS 3.5–7), we performed one-way ANOVA (*p* = 0.0130) and obtained statistical significance between Log LTL healthy controls vs. severe disability (*p* = 0.0094) and mild-moderate disability vs. severe disability (*p* = 0.0140). See Figure 1. There was no significant difference in the frequency of treatments of both RRMS groups, and there was no association of drugs with LTL and mtDNA-CN (Kruskal–Wallis), *p* = 0.193 and 0.471, respectively.

### 2.3. LTL and mtDNA-CN Correlation

**Figure 2:**
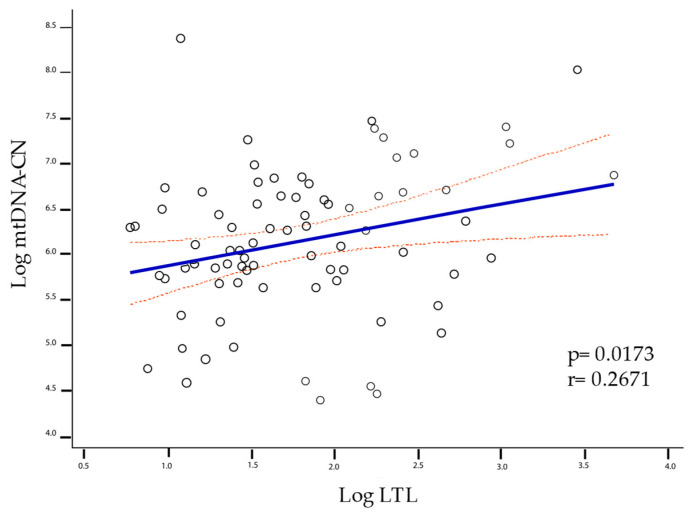
Linear correlation between LTL and mtDNA-CN was significant for all groups (*p* = 0.0173, r = 0.2671). The solid line represents the regression line, and the dashed lines represent the confidence interval of 95%.

**Figure 3:**
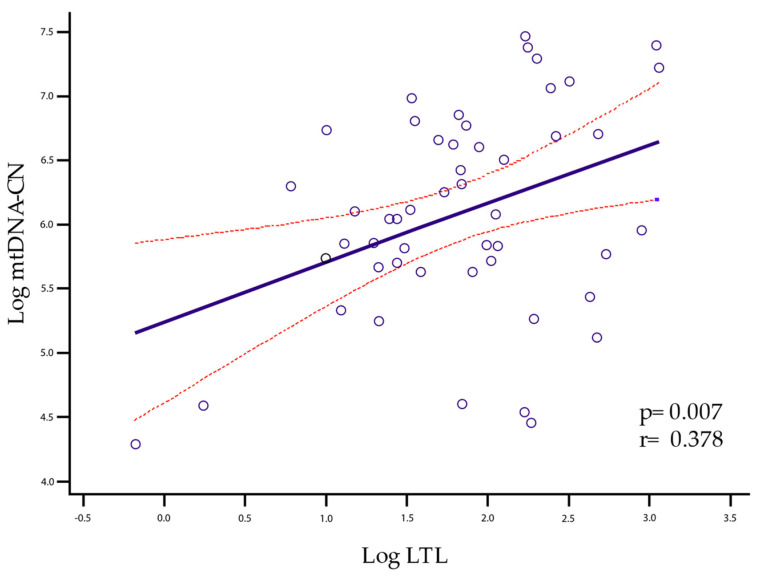
Linear correlation between LTL and mtDNA-CN was significant for the mild-moderate group (*p* = 0.007, r = 0.378). The solid line represents the regression line, and the dashed lines represent the confidence interval of 95%.

The correlation of LTL with mtDNA-CN is given when all individuals are grouped into a single cluster (healthy controls, mild-moderate disability, and severe disability) for which the Pearson test was performed, yielding a *p* = 0.0173, r = 0.2671 (Figure 2). However, when analyzing group by group, significance was only maintained in the mild-moderate group with *p* = 0.007 and r = 0.378 (Figure 3).

**Figure 4:**
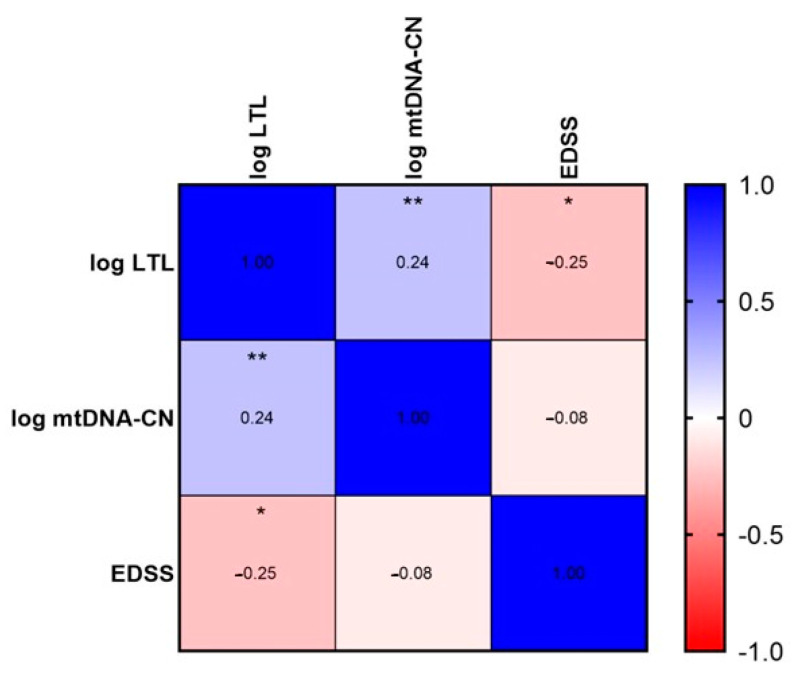
Spearman correlation test was performed with a * *p* = 0.034 for LTL and EDSS and a ** *p* = 0.038 for LTL and mtDNA-CN.

On the other hand, all patients with RRMS grouped together showed a significant correlation in LTL with EDSS and LTL with mtDNA-CN (Spearman’s test yielded a *p* = 0.034 for LTL and EDSS and a *p* = 0.038 for LTL and mtDNA-CN) (Figure 4); consistent with correlations found with Pearson’s test between LTL and mtDNA-CN.

### 2.4. Prediction of Disability by LTL

**Figure 5:**
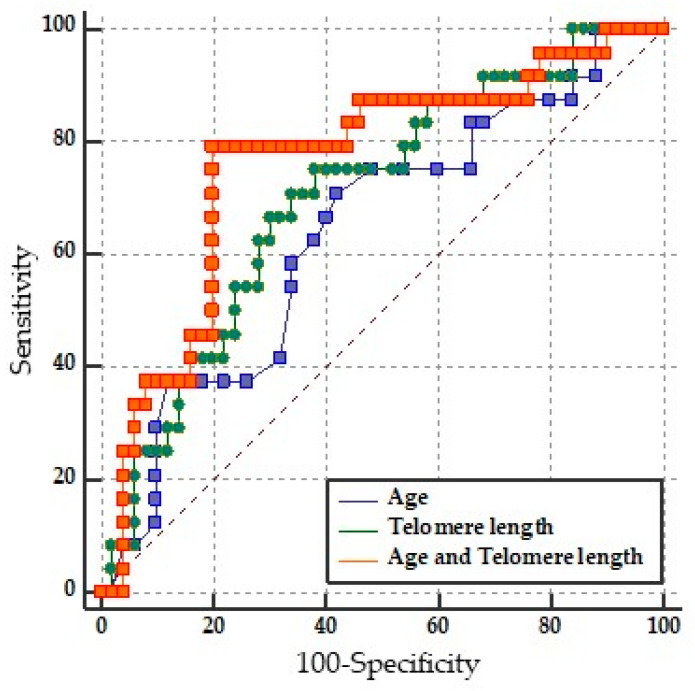
Pairwise comparison of ROC curves of LTL (raw data), age (raw data), and LTL and age (binary logistic regression).

LTL performance in discriminating between mild-moderate disability vs. severe disability was assessed on a ROC curve. An area under the curve (AUC) of 0.702 and a *p* = 0.0018 were obtained. The cut-off point was ≤3.08 Kb, which showed a sensitivity of 75% and specificity of 62%, a PPV of 48.6%, and an NPV of 83.3%. This indicates that LTL alone in the RRMS group can acceptably discriminate between mild-moderately vs. severely disabled patients. See the green line (Figure 5).

### 2.5. Binary Logistic Regression Model of LTL and Age

In the multivariable logistic regression model, only LTL maintained statistical significance when controlling variables in the Wald test, as can be seen in Table 2. When analyzing age and LTL together in the model, discriminative performance improved, which did not occur with other variables (see Table 3).

LTL and age together had the highest precision and were the most appropriate for predicting disability according to the Akaike criteria (AUC = 0.762, *p* = 0.0001, sensitivity = 79.17%, specificity = 80.00%, PPV = 65.5 and NPV = 88.9%). See Table 3. The pairwise comparison of the ROC curves between LTL and age together versus LTL alone did not reach significance (*p* = 0.1452); therefore, the equivalence of the models LTL alone and age vs. LTL was not ruled out, as shown in Table 3. This means that the factor responsible for the predictive ability is LTL, not age.

## 3. Discussion

In recent years, MS has been approached from the perspective of biological senescence. As a trigger for cellular and immunological changes, aging is involved in the development of neuroinflammation and neurodegeneration, resulting in the disability of MS patients. Among the multiple factors that account for neurodegeneration, accelerated neurological aging occurs in MS, and telomeres play an important role in this process. Telomeres are found at the ends of chromosomes and are made up of repeats of the hexanucleotide, non-coding sequence, TTAGGG [15,16]. Changes in telomeres contribute to the pathogenesis of several multifactorial chronic diseases, like neurological disorders. It has been shown that loss of chromosomal integrity due to the shortening of telomere length facilitates fusions with other chromosomes, mutations, and other events that impair cell function, decrease cell division and lead to cell aging [26,27].

On the other hand, mitochondria are organelles that regulate intracellular calcium homeostasis, ATP generation, programmed cell death (apoptosis), ROS production, and aging processes, which together affect the structure and function of telomeres [28]. Additionally, numerical and structural alterations of the mitochondria are pathogenic, especially in tissues with high energy demand, such as muscle and the CNS [29]. Several studies have shown that age and accelerated aging are factors of neurological deterioration in patients with MS. Nevertheless, age is considered an independent factor in developing a progressive MS phenotype through complex and multidirectional interactions with aging and degenerative processes [30,31]. In this sense, telomeres and mitochondria are two of the most important cellular components in regulating aging and neurodegenerative diseases.

The patients included in this study have a mean age and a proportion of mild-moderate/severe disability similar to that observed in the general Mexican population. The wide variability of the LTL and the mtDNA-CN reflects the interactions of the organisms with environmental factors and the influence of endogenous factors like genes, hormones, and ROS, among others. In particular, up to 70% of the variance of the telomere length (TL) is explained by heritability [32,33]. Although the variability in TL is determined by multiple factors, in the group with a mild-moderate disability, we found a significant linear correlation between LTL and mtDNA-CN (*p* = 0.007, r = 0.378). This correlation was also found in the all-pooled participants (healthy controls, mild-moderate disability, and severe disability) (*p* = 0.0173, r = 0.2671). However, when we separated them into distinct groups and analyzed them one by one, only the mild-moderate disability group maintained a significant correlation. This effect could be enhanced or attenuated by other factors related to aging and neurodegeneration processes, such as increased immune activation, chronic inflammation, and age [13,34,35].

The bidirectional interaction between mitochondria and TL, involving different mechanisms related to mitochondrial function (oxidative stress, apoptosis, energy efficiency of the respiratory chain, chronic inflammation), can cause chronic damage leading to telomere shortening. This shortening leads to dysregulation of subtelomeric DNA gene expression [36,37]. As previously described, aging is essential for the progression of MS disability. In fact, senescent changes have been identified in multiple cell lineages of the nervous system that are affected during MS, such as neurons, microglia, oligodendrocytes, and astrocytes [38,39,40,41,42]. In the present study, we compared the absolute quantification of LTL and mtDNA-CN in patients with mild to moderate disability versus patients with severe disability and found significant differences for LTL but not for mtDNA-CN.

Telomere biology and aging play a crucial role in health and disease. In several studies, age has been reported to be one of the most critical factors in converting RRMS to SPMS [43,44,45]. This clinical distinction reflects the underlying neurological damage: in the RRMS phenotype, the damage is predominantly inflammatory, whereas, in the progressive phenotype, it is neurodegenerative [5,46]. Furthermore, LTL can serve as a biomarker of immunosenescence and has also been associated with the progression of neurological damage [44]; Therefore, LTL could be useful for evaluating and predicting disability in patients with MS [47]. In this work, we have found that LTL is an independent predictor of disability. After integrating possible predictors (LTL, mtDNA-CN, gender, age, and time with disease) into the binary logistic regression model, only age and LTL were useful for prediction. The ROC curve for direct LTL values alone has moderate predictive power. The high NPV (83.3%) of LTL identifies it as a relevant biomarker to assess disability status. Although LTL and age are risk factors related to disability, in this study, LTL shortening is the only one that remains an independent factor for MS disability after controlling variables in logistic regression. This is of great importance since age is considered the strongest prognostic factor for MS progression [48]. However, LTL may be as strong or even stronger than age as a predictor of disability status and/or disease progression in MS patients: in the best-performing model (LTL plus age), age may contribute to the cumulative effect of other environmental factors throughout life, even when age is not significant as an independent factor (Table 2).

## 4. Materials and Methods

### 4.1. Study Design and Patient Selection

**Figure 6:**
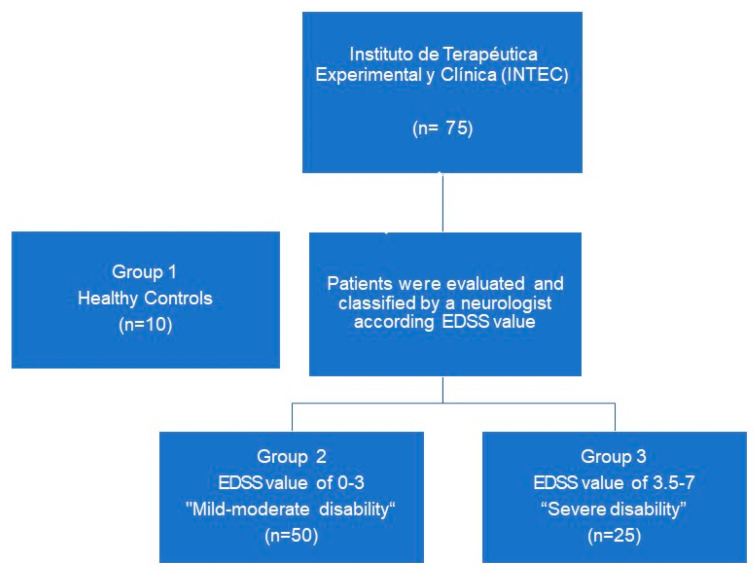
Patient selection flowchart.

This research is a cross-sectional observational study. Seventy-five patients with RRMS were recruited from the MS cohort of the Instituto de Terapéutica Experimental y Clínica (INTEC), of the Centro Universitario de Ciencias de la Salud (CUCS) of the Universidad de Guadalajara from March to October 2021selected (50 women, 25 men, age range: 18–66 years) and 10 healthy controls, in total 85 patients. All patients with RRMS met McDonald’s diagnostic criteria [49], according to the evaluation and diagnosis by a clinical neurologist, and did not present any comorbidities such as cancer, diabetes, hypertension, or other immunological diseases. Patients were divided into three groups, one group as healthy controls, and the other groups were divided according to the RRMS disability: mild-moderate disability, *n* = 50 (EDSS from 0–3), and severe disability, *n* = 25 (EDSS 3.5–7) (Figure 6). In all groups, the parameters of age, time elapsed since disease onset, rate of disease progression, and type of treatment were obtained. This study was conducted in accordance with the Declaration of Helsinki and was approved by the Comité Institucional de ética of CUCS (Study No. CI-03519). Informed consent was obtained from all individual participants included in the study.

### 4.2. Absolute Quantification of Leukocyte Telomere Length (LTL) and Copy Number of mtDNA-CN

Total venous blood samples were collected to isolate leukocytes, and DNA was extracted by the Miller salting out method and stored at −20 °C until analysis [50]. Quantification of mtDNA-CN and measurement of LTL was evaluated by qPCR with the Absolute Human Telomere Length and Mitochondrial DNA Copy Number Dual Quantification qPCR kit (ScienCell Research Laboratories, Carlsbad, CA) according to the manufacturer’s protocol [51].

As mentioned above, LTL measurement and mtDNA-CN quantification were carried out by polymerase chain reaction (PCR). The PCR reaction (per sample) was composed of: 10 μL of 2XGoldNStart TaqGreen qPCR master mix, 2 μL of primer solution (Tel, mtDNA, or a single copy reference, SCR), 7 μL of nuclease-free water, and 1μL of test DNA (5 ng). The conditions of each thermal cycle were carried out as follows: denaturation at 95 °C for 10 min, followed by 32 cycles with denaturation at 95 °C for 20 s, hybridization at 52 °C for 20 s, and extension at 72 °C for 45 s. Finally, a region of 100 bp in length on chromosome 17 was recognized by the first set of SCR and reference DNA with a known concentration of telomere length 348 ± 11 kb per diploid cell and mtDNA-CN of 1.27 ± 0.03 × 10^3^ copies per diploid cell [52].

### 4.3. Statistical Analysis 

Descriptive statistics represented the data of each group. Normality was evaluated using the D’Agostino-Pearson test. The outliners were identified using the Rout method. For comparisons between groups of healthy controls vs. mild-moderate disability vs. severe disability, an ANOVA test was applied for variables with normal distribution; for variables with non-normal distribution, the Mann–Whitney U test was used. The chi-square or Fisher’s exact test was used for qualitative data comparisons. The predictive performance (sensitivity, specificity, and positive and negative predictive value) was evaluated with ROC curves. Binary logistic regression models were developed, and the Akaike information criterion and the Z statistic were used to compare and select models. The cut-off point was obtained through the Youden index. Analyses were performed with GraphPad Prism version 9.02 for Windows (La Jolla, CA, USA; www.graphpad.com (accessed on 12 December 2022)) and MedCalc Statistical Software version 15.8 (Ostend, Belgium; www.medcalc.org (accessed on 12 December 2022)).

## 5. Conclusions

Our study is the first to correlate LTL and mtDNA-CN in RRMS patients with mild to moderate disability. These biomarkers may be useful to unravel the molecular mechanisms involved in the process of neuroinflammation, neurodegeneration, and aging, as well as their relationship with the structure and function of telomeres and mitochondria in MS. However, more studies are required. In particular, longitudinal studies with larger samples are needed to address the different MS phenotypes and thus establish the clinical utility of LTL and mtDNA-CN for assessing and predicting disability.

## Figures and Tables

**Table 1:** Demographic characteristics of RRMS patients and their pharmacological treatments.

Characteristic	Mild-Moderate Disability EDSS 0–3 (*n* = 50)	Severe Disability EDSS 3.5–7 (*n* = 25)	*p*-Value
Female	33 (66%)	17 (68%)	NS
Male	17 (34%)	8 (32%)	NS
Age	37.7 ± 11.4	43.0 ± 11.1	NS
Years since diagnosis	7.78 ± 5.12	10.2 ± 6.99	
EDSS ‡	1.91 ± 0.88	4.9 ± 1.11	* 0.00001
Progression rate	0.51 ± 0.63	0.99 ± 1.09	* 0.00145
Treatment:			
Glatiramer Acetate	19 (38%)	9 (36%)	NS
Rituximab	4 (8%)	3 (12%)	
Interferon β	14 (28%)	5 (2%)	
Fingolimod	5 (10%)	2 (8%)	
Azathioprine	0	3 (12%)	
Natalizumab	1 (2%)	1 (4%)	
Dimethyl fumarate	3 (6%)	1 (4%)	
None	4 (8%)	1 (4%)	

‡ EDSS = Expanded Disability Status Scale. Progression rate = EDSS/years with the disease. Comparisons were performed with the *t*-test, Mann–Whitney test, or χ^2^, as appropriate. * significant, NS (Not Significant).

**Table 2:** The binary logistic regression model evaluated characteristics to predict severe disability in patients with RRMS.

Variable	Odds Ratio	95% CI	*p*-Value
LTL *	0.3458	0.1384 to 0.8641	* *p* = 0.0230
mtDNA-CN	1.0723	0.4792 to 2.3994	*p* = 0.8651
Age	1.0205	0.9686 to 1.0753	*p* = 0.4457
Sex (female)	1.0687	0.3369 to 3.3899	*p* = 0.9102
Years since diagnosis	1.0702	0.9698 to 1.1810	*p* = 0.1771

LTL: Leukocyte telomere length; mtDNA-CN: Mitochondrial DNA copy number. * *p* = 0.0230, Wald’s test. CI: Confidence interval.

**Table 3:** Model comparison analysis.

Variable	AUC	95% CI	*p*-Value
Age	0.635	0.516 to 0.743	* *p* = 0.0488
LTL	0.702	0.584 to 0.803	* *p* = 0.0018
LTL and Age	0.762	0.649 to 0.854	* *p* = 0.0001
**Pairwise comparison of ROC curves**			
Age vs. Age + LTL		0.0122 to 0.231	* *p* = 0.0294
LTL vs. Age + LTL		−0.0210 to 0.143	*p* = 0.1452
Age vs. LTL		−0.113 to 0.235	*p* = 0.4925

LTL: Leukocyte Telomere Length; AUC: Area under the ROC curve. Age, LTL, and LTL and age with significance: * *p* = 0.0488, * *p* = 0.0018, and * *p* = 0.0001. Pairwise comparison of ROC curves was significant only in Age vs. Age + LTL * *p* = 0.0294.

## Data Availability

https://figshare.com/articles/dataset/TLT_mtDNA_MS/17097470 (accessed on 12 December 2022).
